# Local Structure Analysis and Modelling of Lignin‐Based Carbon Composites through the Hierarchical Decomposition of the Radial Distribution Function

**DOI:** 10.1002/open.202100220

**Published:** 2022-02-17

**Authors:** Dayton G. Kizzire, Valerie García‐Negrón, David P. Harper, David J. Keffer

**Affiliations:** ^1^ Materials Science and Engineering Department University of Tennessee, Knoxville 1508 Middle Dr Knoxville TN 37996 USA; ^2^ Sustainable Biofuels and Co-Products Research Unit USDA – Agricultural Research Service/Eastern Regional Research Center 600 East Mermaid Lane Wyndmoor PA 19038 USA; ^3^ The Center for Renewable Carbon – UT Institute of Agriculture University of Tennessee, Knoxville 2506 Jacob Dr Knoxville TN 37996 USA

**Keywords:** composite materials, graphite, lignin, modelling, sustainable

## Abstract

Carbonized lignin has been proposed as a sustainable and domestic source of activated, amorphous, graphitic, and nanostructured carbon for many industrial applications as the structure can be tuned through processing conditions. However, the inherent variability of lignin and its complex physicochemical structure resulting from feedstock and pulping selection make the Process‐Structure‐Property‐Performance (PSPP) relationships hard to define. In this work, radial distribution functions (RDFs) from synchrotron X‐ray and neutron scattering of lignin‐based carbon composites (LBCCs) are investigated using the Hierarchical Decomposition of the Radial Distribution Function (HDRDF) modelling method to characterize the local atomic environment and develop quantitative PSPP relationships. PSPP relationships for LBCCs defined by this work include crystallite size dependence on lignin feedstock as well as increasing crystalline volume fraction, nanoscale composite density, and crystallite size with increasing reduction temperature.

## Graphical abstract

**Radial distribution functions** from X‐ray and neutron scattering of lignin‐based carbon composites (LBCCs) are investigated to characterize the local atomic environment and develop quantitative processing‐structure‐property‐performance relationships for the optimization of lignin‐based hard carbon anodes for Li and Na‐ion batteries. Modelling and analysis are completed with the Hierarchical Decomposition of the Radial Distribution Function.

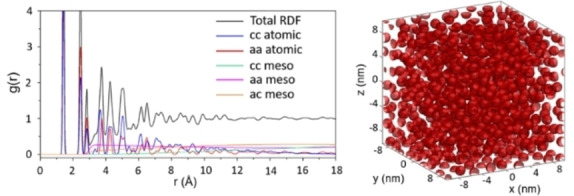

## 1 Introduction

Since today's energy market is focused on reducing pollution and providing more efficient and sustainable energy storage devices, energy technologies manufactured with bio‐based and sustainable materials are necessary.^[1]^ The incorporation of bio‐based materials into energy technologies comes with significant challenges, usually due to their often disordered and complex nature compared to their inorganic counterparts. Recently, researchers have developed methods to tune these features to their advantage and have made significant strides in the development of nanostructured and bio‐based materials for cathodes,^[2]^ electrolytes,[^2b^, ^2c^, ^3^] anodes,^[4]^ super capacitors,^[5]^ and fully organic batteries,^[6]^ resulting in the development of safer, longer lasting, and higher charge density batteries, super capacitors, and fuel cells for use in electric vehicles, mobile electronics, large scale grid applications, and so forth.^[7]^

A primary area of interest lies in generating a sustainable and domestic source of carbon for the energy, manufacturing, and technology industries. Lignin is an amorphous, cross‐linked structure of aromatic polymers derived from woody plants and grasses and is the world's largest source of renewable carbon. Recent research has stated that lignin can be used to manufacture high quality carbon composites that can be tuned through processing conditions for use in lithium/sodium battery anodes and super capacitors.[^4a^, ^5^, ^8^] The structure and properties of carbonaceous products manufactured from lignin result from the choice of processing conditions and the relative percentages of the primary monomeric units (syringyl, guaiacyl, and p‐hydroxyphenyl) that constitute lignin.^[9]^ However, developing a predictive processing – structure – property – performance (PSPP) relationship between lignin and carbonaceous products presents a significant challenge given that the relative fractions of monomeric units are highly variable between feedstocks and that the environmental and processing conditions largely effect the resultant structure and properties. These factors combined make developing a predictive PSPP relationship a complex process and increases the difficulty of manufacturing a consistent nanostructured material from amorphous precursors.

Previous research has found that carbonization of lignin produces a heterogeneous two‐phase carbon composite comprised of nanoscale graphitic domains and domains of randomly oriented amorphous graphene fragments.^[9]^ The structure and properties of this two‐phase carbon composite is highly dependent upon reduction temperature with reduction temperatures near 1000 °C producing a mostly amorphous graphene composite with small graphitic domains and temperatures near 2000 °C producing large graphitic domains embedded in an amorphous graphene matrix.^[9]^ Anodes synthesized from lignin‐based carbon composites (LBCCs) reduced at 1050 °C have delivered a charge capacity of 444 mAh/g with coulombic efficiency of 98 % over extended galvanostatic cycles in Li‐ion coin cell batteries.^[4a]^ Recent simulation work has found that lithium and sodium storage mechanisms are fundamentally different in LBCCs and has suggested that LBCC anodes in sodium ion batteries would possess high charge capacities, high cycling performance, and high diffusion rates due to the unique nanostructure of LBCCs.^[10]^ In order to optimize LBCCs for performance as sustainable anodes and across multiple technologies, we require unambiguous knowledge of the nanostructure. Previous work with lignin and LBCCs has revealed the relative fractions of monomeric units present in each feedstock and further characterization experiments using TEM, XRD, Raman, XPS, and NMR have developed excellent qualitative information on the LBCC nanostructure as well as trends in PSPP relationships between reduction temperature and nanostructure.^[9]^ However, the detailed quantitative information on LBCC local structure such as phase volume fraction, phase density, particle shape, and particle size are only attainable through a combination of modeling and experiment.

Traditionally, determining the local structure of complex nanomaterials with large amorphous components is accomplished through the hypothesis of a model structure based on experimentally observed features and simulation using large‐scale molecular dynamics (MD) to capture the mesoscale structure of the material. Since the radial distribution function (RDF) is an effective function for evaluating the local structure of powder, single‐crystal, or liquid materials containing amorphous or crystalline domains in isotropic or anisotropic orientation,^[11]^ model analysis usually includes comparison of the experimental neutron or X‐ray RDFs and the simulated RDF.^[12]^ This method allows researchers to directly attribute a complex nanomaterial's structural characteristics to features present in the simulated RDF,[^11^, ^13^] and in battery specific research it can define local order changes from cycling, nano‐phase quantifications, and ion storage mechanisms.[^9^, ^10b^, ^12^, ^13b^, ^14^] While this method is effective for testing specific composites, it produces a bottleneck when researching materials where small changes in processing have large effects in the resultant structure and the subsequent performance of the material in application. Such problems would be better solved with a process where the model's structural parameters are refined iteratively; however, this is impractical with MD simulations as complex nanomaterials are generally computationally expensive due to the large system sizes required to capture the nano and meso‐scale order.^[15]^ This problem presents the need for a computational tool to quickly model and iteratively refine complex nanostructured materials without a severe computational cost.

Although there are many, some of the current endeavors in developing a generalized tool for structural analysis of complex materials include the Diffpy‐Complex Modelling Framework, the TOPAS‐Academic software package, DISCUS, and RMCprofile.^[16]^ Due to the size of model needed to accurately capture the mesoscale order of LBCCs, the fact that every clear RDF peak arises from a plane of graphene, and the inherent complex nature of LBCCs, it was deemed necessary to employ a different modelling approach to accurately determine the local and mesoscale structure of LBCCs. In 2016 Oyedele et al. proposed a novel, physics‐based model for RDF studies known as the hierarchical decomposition of the radial distribution function (HDRDF) method where atomistic and mesoscale models and theory are combined to construct the total RDF without arbitrary fitting parameters.^[17]^ This method of modelling also allows researchers to distinguish features in the RDF from ordered or disordered domains of a complex, multiphase material. The aim of HDRDF is to fill a need in the scientific community for a quick and computationally efficient method to determine the local structure of complex nanomaterials. HDRDF 3 was created to address the major needs of previous versions and incorporate arbitrary domain geometries, mesoscale (a)symmetry, and automated parameter optimization. A thorough explanation of the theory and process by which HDRDF 3 generates RDF models and iteratively determines structural features can be found in the experimental section. In this work we employ HDRDF 3 together with TEM images and synchrotron X‐ray RDFs to determine the crystalline and amorphous particle shapes and sizes, component volume fractions, as well as component and composite densities for an array of LBCCs synthesized from four unique lignin feedstocks processed under a range of reduction temperatures. This accurate description of the local structure allows us to quantitatively define the PSPP relationships of lignin and further develop sustainable carbonaceous products.

## 2 Results and Discussion

### 2.1 Model Validation

**Figure 1:**
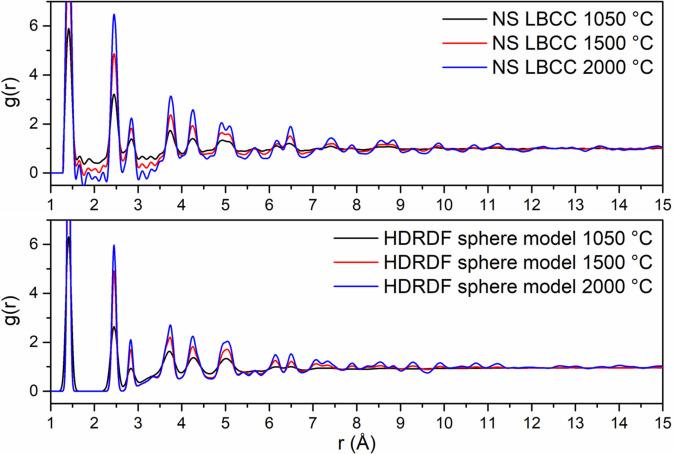
(Top) Neutron scattering RDFs of hardwood LBCCs synthesized by Tenhaeff et al.^[8]^ with increasing carbonization temperature. (Bottom) HDRDF 3 modelled RDFs of carbon composites.

In order to validate HDRDF 3 (henceforth referred to as HDRDF), as well as showcase the increased accuracy and functionality of this iteration of HDRDF, we apply it to neutron scattering (NS) RDF data of three hardwood derived carbon composites shown in Figure 1. The NS data used for HDRDF model validation was previously gathered and analyzed via MD by McNutt et al., experimentally characterized by Tenhaeff et al., and modeled with a previous version of HDRDF by García‐Negrón et al.[^13a^, ^17^, ^18^] A systematic analysis of crystalline domain shape, size, and volume fraction was conducted for the three samples, where crystallite size and crystalline volume fraction were varied for right parallelepiped, rod, sphere, and ellipsoid particle shapes and compared for best fit to the neutron scattering RDF data. Results from this analysis agreed well with the structural parameters found in the previous version of HDRDF published by García‐Negrón et al., which showed the best model for this data uses spherical particles with increasing particle radius and decreasing crystalline volume fraction with the increasing carbonization temperature of the three carbon composites.^[18]^ The RDFs for the three composites with their respective HDRDF models are shown in Figure 1 below with the optimized structural parameters shown in Table 1. We can see from Figure 1 that the magnitude of the peaks in the HDRDF model are consistent with the peak magnitudes from NS experiments. Since all peak positions are represented by HDRDF, it confirms that the graphene fragments used to model the atomic contribution for the amorphous phase are correct; if the amorphous phase contained *sp* ^*3*^ bond hybridization then peak positions in the HDRDF model would not match the NS experiments. The density for the crystalline and amorphous domains were input as 2.26 and 0.95 g/cm^3^ respectively, consistent with literature values for crystalline graphite and both 2D and 3D amorphous graphene with *sp* ^*2*^ bonding.^[19]^ It is important to note that the HDRDF modeled RDFs are calculated directly and thus have no short or long‐range oscillations (Fourier ripples) that arise from the Fourier transform used to convert the experimental scattering function *S(Q)* to the RDF, and contains no artifacts from equipment effects or sample inhomogeneity as occurs in experimentally obtained RDFs. This implies that every peak in a HDRDF modeled RDF arises due to material structure. It should also be noted that the peak widths of RDFs modeled with HDRDF are slightly narrower than the experimental comparisons due to peak broadening that occurs for several reasons, including ball milling of graphitic structures.^[20]^ In order to give a more accurate measure of agreeability between the NS data and HDRDF models, the mean absolute error reported in Table 1 was calculated for *r* between 3.59 and 20.0 Å as to not include error from Fourier ripples present below 3.59 Å.

**Table 1:** Optimized structural features for Hardwood LBCCs synthesized by Tenhaeff et al.^[8]^

Optimization Parameters	Previous Model			HDRDF 3		
Reduction Temperature [ °C]	1050	1500	2000	1050	1500	2000
Crystallite Shape	Sphere	Sphere	Sphere	Sphere	Sphere	Sphere
Crystallite Radius [Å]	5	7	17	5	7	17
Crystalline Volume Fraction [%]	90	50	10	85	50	20
Composite Density [g/cm^3^]	1.94	1.51	1.38	2.07	1.61	1.21
Graphene Fragment Major Radius [Å]	2.5	24	15	5	15	15
Graphene Fragment Minor Radius [Å]	2.5	4	15	3	5	15
Intraplanar Thermal Noise [Å]	0.025	0.025	0.025	0.030	0.030	0.030
Interplanar Thermal Noise [Å]	n/a	n/a	n/a	0.050	0.050	0.050
Mean Absolute Error (MAE) (3.59<r<20.0 Å)	n/a	n/a	n/a	0.061	0.074	0.089

### 2.2 Modelling Carbon Composites

**Figure 2:**
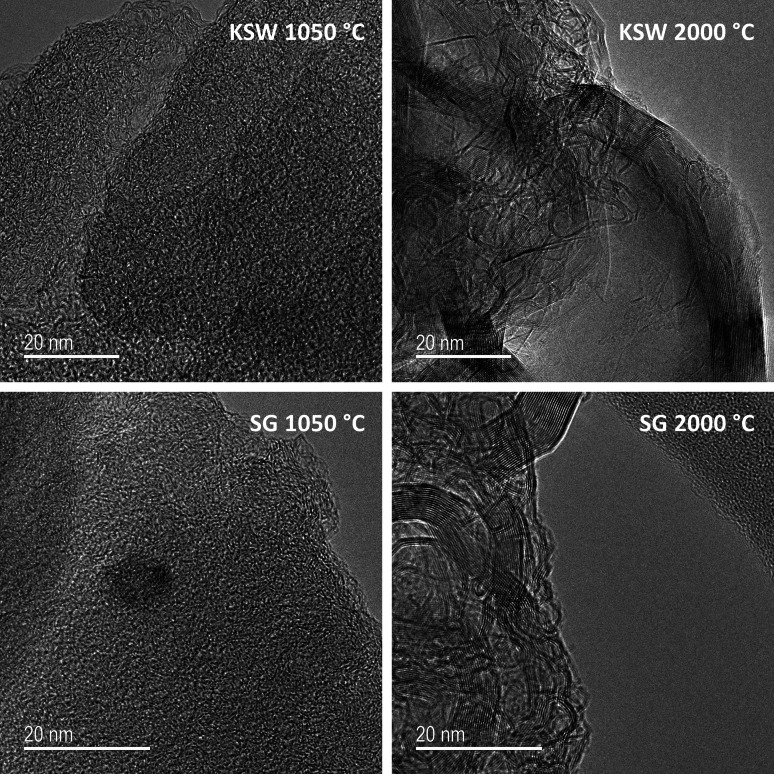
HR‐TEM images of hard carbon composites produced from kraft softwood (KSW) and switchgrass (SG) lignin feedstocks with reduction temperatures of 1050 and 2000 °C.

**Figure 3:**
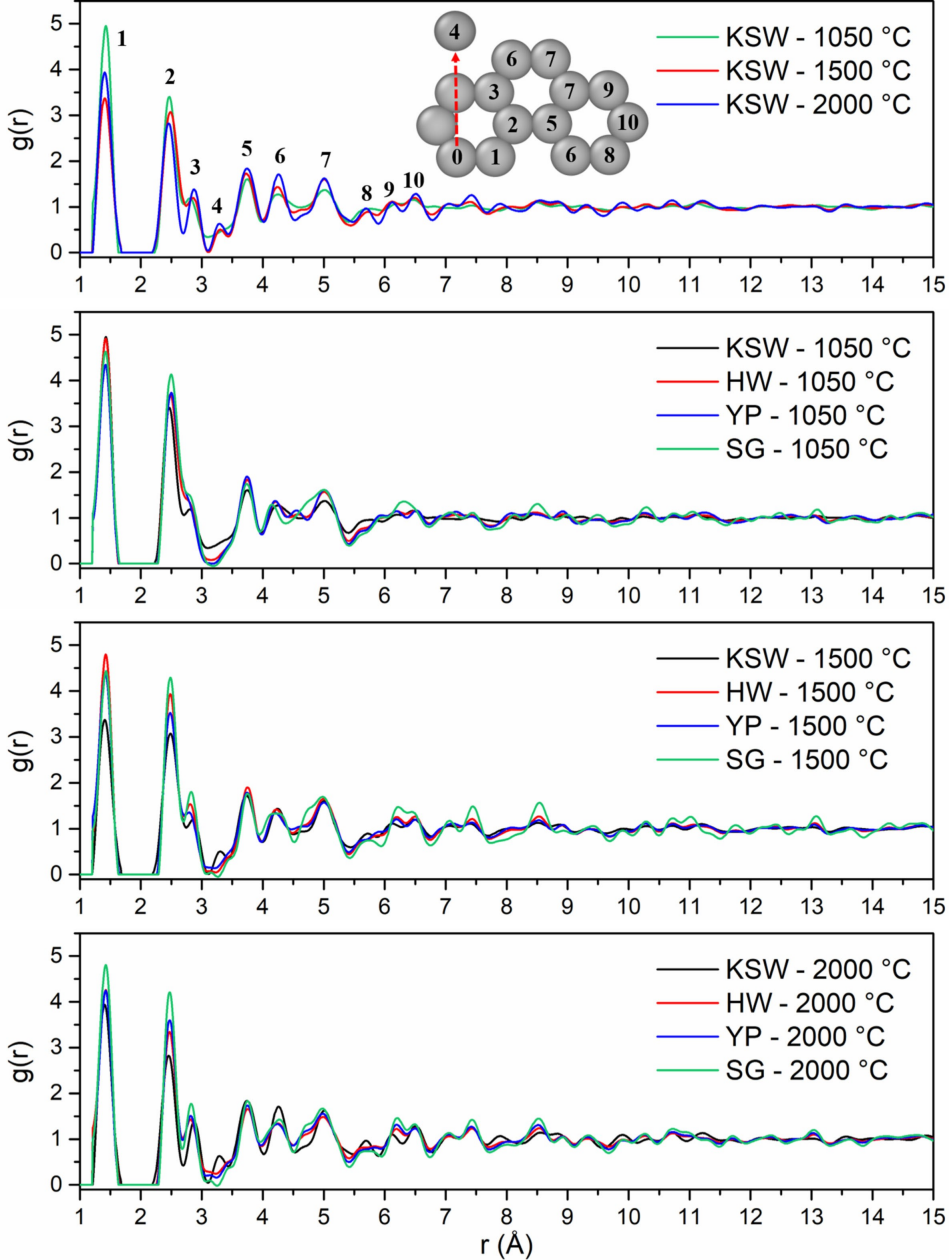
Synchrotron X‐ray RDFs of LBCCs grouped by carbonization temperature. (Top) Diagram identifying atomic pairs and the peak to which they correspond as measured from Atom 0. Atom 0 to Atom 4 represents the interplanar spacing of graphitic planes.

**Figure 4:**
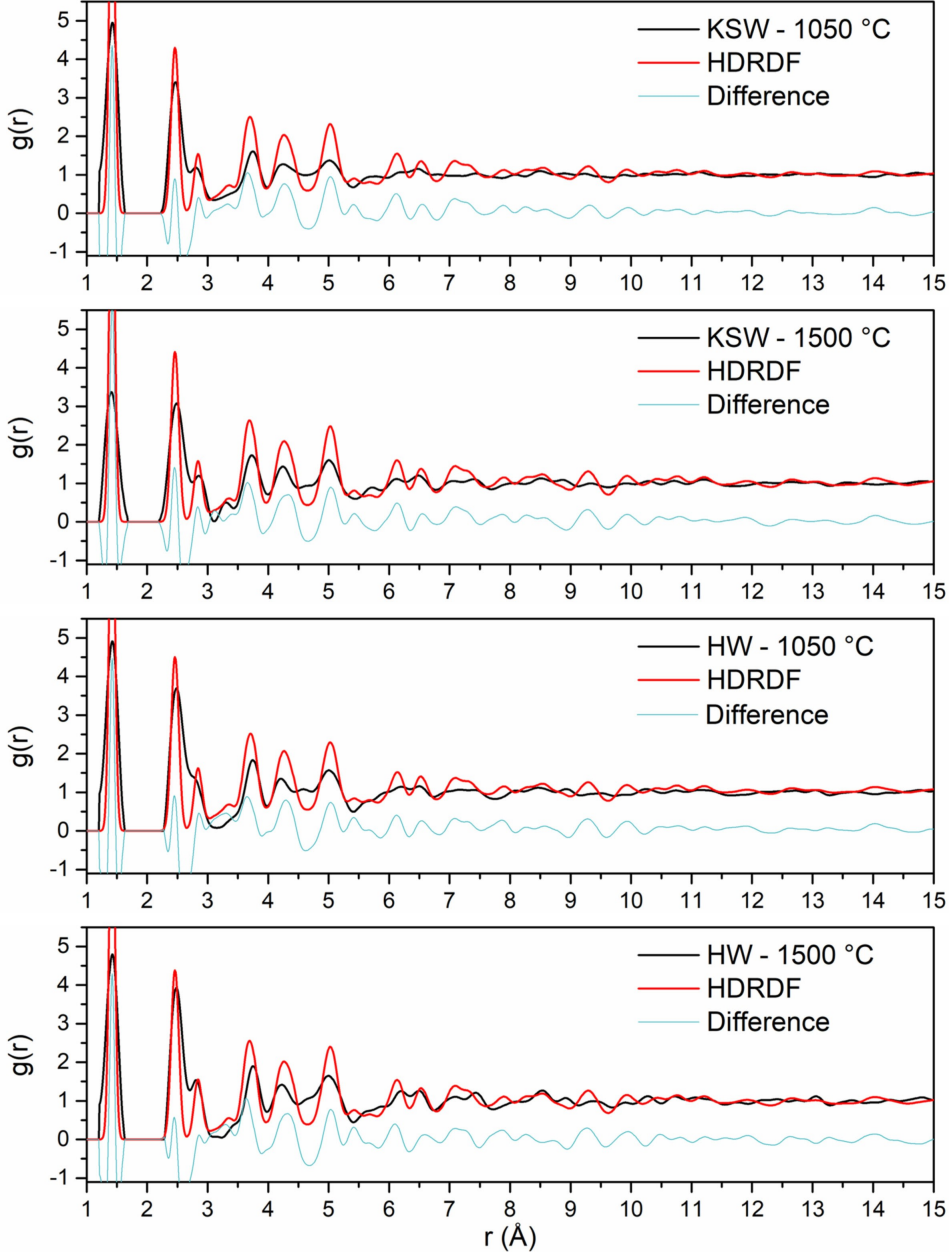
Synchrotron X‐ray RDFs of LBCCs reduced at 1050 and 1500 °C plotted with their respective HDRDF models.

**Figure 5:**
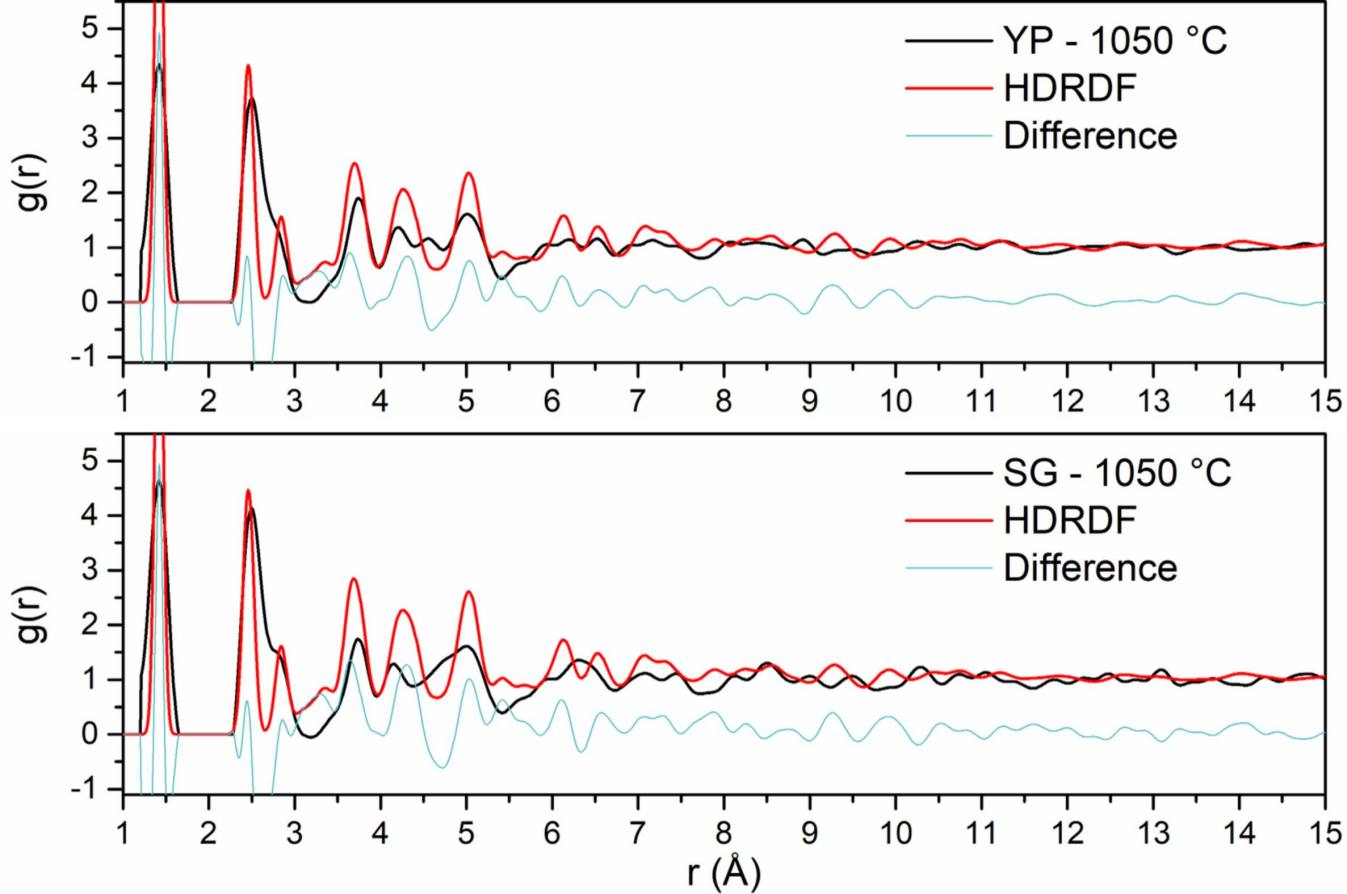
Synchrotron X‐ray RDFs of LBCCs reduced at 1050 °C plotted with their respective HDRDF models.

The remainder of this work models synchrotron X‐ray RDF data of LBCC samples synthesized and experimentally analyzed by García‐Negrón et al..^[9]^ It is important to note that the crystallites in the LBCC samples synthesized by García‐Negrón et al.^[9]^ are slightly more than an order of magnitude larger than the crystallites in the Hardwood LBCCs synthesized by Tenhaeff et al.^[8]^ which were used for HDRDF model accuracy verification. The size difference in crystallite domains can be attributed to differing lignin feedstock, synthesis methods, and post‐synthesis ball milling procedure. From visual inspection of the TEM images in Figure 2 and the 1050, 1500, and 2000 °C RDFs in Figure 3, it is evident that the local structure of the carbon composites is not only dependent upon carbonization temperature but also lignin feedstock. The RDFs of the woody species of lignin feedstocks, including kraft softwood (KSW), organosolv Southern yellow pine (YP), and organosolv hardwood (HW), share greater similarities, relative to the organosolv switchgrass (SG) samples, which have a comparably different structure for the 1000 and 1500 °C samples. This differing local structure can be attributed to the varying concentrations of p‐hydroxyphenyl (H), guaiacyl (G), and syringyl (S) phenolic units that compose the cross‐linked, amorphous structure of lignin. The LBCCs increase in crystallinity as reduction temperature is increased and the 2000 °C samples show the greatest similarity implying the structures have become more graphitic in nature. The third (2.87 Å) and fourth (3.29 Å) peaks represent the third nearest neighbor and interlayer spacing respectively as shown in the diagram in Figure 3. The evolution of the third peak from a shoulder to a distinct peak shows the transformation of the mostly disordered amorphous carbon composite to a more graphitic C_6_ type structure. The stark increase in distinction of the fourth peak for 1500 and 2000 °C conveys that the carbon composite structure becomes more graphitic as planes of graphene grow and align into their equilibrium interplanar distance. Further, the increasing peak intensity past 7 Å for each increase in reduction temperature denotes longer range order implying increased crystallinity. To reveal more about the local structure other than trends in crystallinity, we turn to modelling the carbon composites with HDRDF, with comparisons shown in Figures 4 and 5, and HDRDF optimized structural features shown in Table 2.

**Table 2:** Structural features of LBCCs calculated through HDRDF.

Reduction Temperature	1050 °C				1500 °C	
Lignin Feedstock	KSW Kraft Softwood	HW Hardwood	YP Yellow Pine	SG Switchgrass	KSW Kraft Softwood	HW Hardwood
Crystallite Shape	Sphere	Sphere	Sphere	Sphere	Ellipsoid	Ellipsoid
Crystallite Radius [Å] (x,y,z)	22	23	24	28	28, 24, 32	33, 30, 42
Crystalline Volume Fraction [%]	20	15	20	20	45	40
Composite Density [g/cm^3^]	1.94	1.92	1.94	1.81	2.04	2.02
Graphene Fragment Major Radius [Å]	10	12	14	16	18	21
Graphene Fragment Minor Radius [Å]	8	8	11	12	14	13
Intraplanar Thermal Noise [Å]	0.04	0.04	0.04	0.04	0.04	0.04
Interplanar Thermal Noise [Å]	0.06	0.06	0.06	0.06	0.06	0.06
Mean Absolute Error (MAE)	0.063	0.073	0.076	0.098	0.093	0.098

It is also important to note that there are peaks in the experimentally obtained data that do not correspond to graphite or any of its allotropes and have been confirmed through elemental analysis as varying amounts of oxygen from ether linkages that persisted through pyrolysis as well as iron contamination from the ball milling process.^[9]^ Since we did not include models in HDRDF for the contaminants, the modeled RDFs do not perfectly fit the experimental data. However, there is still much qualitative and quantitative information to be gleaned from the model that include shape and size for crystalline domains and the amorphous graphene fragments, component volume fractions, composite densities, and how trends in these structural features can aid in the understanding of the PSPP relationships. Optimized structural features for each model can be found in Table 2.

### 2.3 Particle Shape and Size

HDRDF models were made for all samples with a reduction temperature of 1050 °C as well as the KSW and HW samples reduced at 1500 °C. The remaining samples with reduction temperatures of 1500 and 2000 °C possessed crystalline domains greater than 140 Å. Since these crystalline domains are much larger than the experimental RDF length of 50 Å, no meaningful crystallite shape analysis could be conducted with HDRDF and they are not modeled in this work. Experimentally, it is well established that an increase in reduction temperature leads to a corresponding increase in size of the graphitic nanocrystallites.^[9]^ Experimental evidence regarding the relationship between nanocrystallite shape and reduction temperature is less clear. However, the TEM work of García‐Negrón et al. suggests that the larger graphitic nanocrystallites that appear at high reduction temperatures are more likely to contain distinctly non‐spherical geometry, presumably due to anisotropic growth of graphite in the directions parallel (100 and 010) and normal (001) to the stacked sheets. To our knowledge there is limited understanding of how choice of lignin feedstock impacts crystallite size. García‐Negrón reports two nuanced observations in this regard. First, principal component analysis of RDFs suggests that differences in LBCC local structure, resulting from variation in the distribution of lignin monomers in the source plant, tend to disappear as the reduction temperature is increased. In other words, all lignin materials will eventually form an increasingly graphitic structure if the temperature is sufficiently high. Second, differences in the size of the resulting crystallites are most obvious at the highest reduction temperatures, with KSW and SG yielding larger crystallites than HW and YP.^[9]^ We make a third observation upon review of García‐Negrón's elemental analysis of the “other” column for pyrolyzed and reduced lignin, where the “other” column is strongly considered to be mainly oxygen from ether linkages with trace amounts of ash and iron contamination from pyrolysis and ball milling respectively.^[9]^ Evidence for ether linkages persisting post pyrolysis is present in the experimental RDFs as there is a relatively high intensity plateau centered near 4.6 Å that decreases in intensity with increasing reduction temperature. This plateau can be observed between peaks 6 and 7 in the KSW comparison in Figure 3 and is present in varying degrees for the other feedstocks as well. Since the all‐carbon HDRDF models produce a low intensity valley in the model RDF near 4.6 Å (visible in Figure 4) we can infer that this feature arises from a source other than the crystalline and amorphous graphene domains. The interatomic distances of an array ether linkages and lignin monomeric units were reviewed and the very common β‐O‐4 ether linkage, which accounts for a minimum of 50 % of all inter‐monomeric linkages in lignin,^[21]^ has a carbon‐carbon separation in the range of 4.56–4.67 Å in the carbon‐oxygen‐carbon ether linkage according to the molecular modelling work performed by Besombes et al., which would account for the high intensity plateau centered at 4.6 Å.^[22]^ The decrease in intensity of this region as reduction temperature increases is consistent with the dissociation of ether linkages at higher reduction temperatures and confirms that some ether linkages persist through reduction at 1050 and 1500 °C. Additionally, given that there are only trace amounts of contamination in all samples, simple subtraction between feedstocks in the “other” column shows that there is at least 25–50 % more oxygen in the KSW and SG samples reduced at 1050 °C compared to the HW and YP samples.^[9]^ We suggest that the greater amount of ether linkages in the KSW and SG samples provide a degree of order and serve as a scaffold along which the graphitic crystallites can grow larger at an increased rate as reduction temperature increases. The large graphitic crystallites of KSW and SG samples reduced at 2000 °C can be seen in the HR‐TEM images in Figure 2 and the considerably smaller crystallites of HW and YP samples reduced at 2000 °C can be found in HR‐TEM images of García‐Negrón et al.^[9]^

A systematic shape and size analysis was conducted for each of the modeled composites where sphere, ellipsoid, rod, and right parallelepiped shapes were tested and the dimensions for each shape were optimized via conjugate gradient (CG) optimization and the resulting RDFs were compared for best fit via least squares error between the experimental and modeled RDFs. Since modelled peaks at low radial distances (below 10 Å) are narrower and taller than experimental peaks due to instrumental peak broadening and inherent sample inhomogeneity/disorder not captured by HDRDF, a weighting function was applied to the least squares error calculation which emphasized the differences at longer radial distances (above 10 Å) in order to help determine particle shape and size more accurately. In Table 2, the mean absolute error between the data and models is reported. All samples reduced at 1050 °C possessed spherical particle shapes consistent with validation data of smaller crystallites from previous neutron scattering experiments. The modeled spherical crystallites for the 1050 °C samples ranged from 4.4 to 5.6 nm in diameter depending on the feedstock. As the reduction temperature increased, the HDRDF analysis confirms growth of the crystallite size and an increase in crystalline volume fraction. Furthermore, the shape of the crystallites deviates from spherical. The 1500 °C samples were best fit with prolate ellipsoidal crystallites with the interplane direction acting as the major radius of 3.2–4.2 nm and the in‐plane directions acting as minor radii of 2.4–3.3 nm. As reduction temperature is increased the graphene planes align and equilibrate into an interplanar distance of 3.35 for KSW and 3.44 nm for all other samples as can be seen by the examination of the fourth peak in the experimental RDFs in Figure 3 as well as the HDRDF fits in Figures 4 and 5. The adoption of surrounding amorphous planes of graphene into crystallites contributes to the change in crystallite shape from spheres to ellipsoids. The modeled crystalline domain sizes are in good agreement with the Scherrer analysis performed on the scattering data by García‐Negrón et al.^[9]^ Amorphous graphene fragments with circular and elliptical shapes were tested with the result of 2D ellipses having the better fit. The 2D ellipses possessed smaller major and minor radii than the crystallites, consistent with previous models and our physical understanding of the composite.

The HR‐TEM of KSW and SG samples reduced at 2000 °C in Figure 2 show primarily crystalline graphitic domains with large polygonal onion‐like nanocrystallites, as well as large, elongated rod like structures that could be multi‐walled carbon nanotubes or collapsed carbon nanotubes based on similarities in TEM patterns found in literature.[^9^, ^23^]

### 2.4 Crystalline Volume Fraction

From visual inspection of the HR‐TEM images reported by García‐Negrón et al.^[9]^ and the images in Figure 2, there is a definite increase in the crystalline volume fraction for each feedstock with increasing reduction temperature. Samples reduced at 1050 °C show a primarily amorphous structure with small amounts of nanocrystallites while samples reduced at 2000 °C show primarily graphitic and ordered structures which are most easily observed in the kraft softwood and switchgrass samples. Nanocrystallites in the pine and hardwood samples reduced at 1050 °C and 2000 °C are somewhat difficult to make out visually; however, the XRD and Scherrer analysis confirm their presence with new peaks forming in the XRD pattern as reduction temperature is increased.^[9]^

HDRDF models for the 1050 °C samples range from 15 % crystalline volume fraction for hardwood to 25 % crystalline volume fraction for switchgrass. Models for the 1500 °C samples found an increase in crystalline volume fractions up to 45 %. These results agree well with the HR‐TEM and XRD – Scherrer analysis conducted by García‐Negrón et al.;^[9]^ however, they are in partial disagreement with the trends modeled by McNutt et al.^[13a]^ who states that for the LBCCs synthesized from hardwood lignin by Tenhaeff et al.,^[8]^ crystalline volume fraction decreases with increasing reduction temperature. They observed larger crystallites at higher reduction temperatures, but a lower total crystalline volume fraction.

### 2.5 Composite Density

Results from analysis of Figure 2 and the HR‐TEM and X‐ray diffraction analysis conducted by García‐Negrón et al. show an increase in graphitic structure as well as a reduction in amorphous regions with increasing reduction temperature for all feedstocks.^[9]^ This would suggest a monotonic increase in the local composite density at the nanoscale with increasing reduction temperature; however, since the composite densities were not determined experimentally there is a degree of uncertainty. For HDRDF modeled composites the density for the crystalline and amorphous domains were input as 2.26 and 1.76 g/cm^3^ respectively, except for the switchgrass sample reduced at 1050 °C which was better fit with an amorphous phase density of 1.69 g/cm^3^. The amorphous carbon density was found to be greater in the models for the García‐Negrón et al. composites when compared to the amorphous carbon density of the composites synthesized by Tenhaeff et al. We believe that the difference in the modeled amorphous phase density between the Tenhaeff et al. composites and the García‐Negrón et al. composites can be attributed to the differences in the used feedstocks, as well as the differences in processing and carbonization process of the lignin. As reduction temperature increased the modelled composite density also increased towards the density of crystalline graphite as would be expected with a larger crystalline volume fraction. The reported composite densities in Table 2 are likely slightly overestimated since porosity and sample packing density present in experimental samples is not captured by the model. In future updates to the HDRDF software, we plan to improve this area by including customizable options for various states of porosity in the mesoscale model.

### 2.6 HDRDF 3 Limitations

As with many other modelling techniques, HDRDF 3 has limits on the size of a system that it can model effectively. For HDRDF 3 the limit is dependent upon the length of the experimental RDF and the size of the crystalline domains. Since the RDF is used for local structure determination, if the average particle size is much greater than the length of the experimental RDF accurate modelling becomes difficult. When modelling nanomaterials with HDRDF 3, the peak heights, widths, and mesoscale features of modeled RDFs are sensitive to changes in particle size and component volume fractions; however, when crystallites have domains greater than nanoscale size, the RDFs no longer contain the information which would allow the determination of particle size or shape and the modeled RDFs resemble multiphase bulk materials instead of nanoscale composites as it is in our case for the composites reduced at 2000 °C as well as the SG and YP samples reduced at 1500 °C.

As currently written, HDRDF contains only two magnitudes of thermal noise, one for pairs of atoms within the same plane and a second for pairs of atoms in different planes. Because the thermal ellipsoid depends upon the local environment, the thermal noise is properly a function of the atomic potential around the equilibrium position. While there are no atomic potentials in HDRDF, molecular dynamics simulations have shown greater configurational disorder as the size of the nanocrystallite decreases.^[15]^ Therefore, it is conceivable to implement thermal broadening as an empirical function of distance from the edge of the crystallite, where the noise approaches a bulk value for sufficiently large crystallites. This feature is not explored in this work because of the overall disorder of the system. There are not sufficiently accurate targets to justify the additional parameters needed to create the configurational thermal noise function.

In the comparison of simulated and experimental RDFs, very modest changes in atomic coordinates can create pronounced changes in the RDF, including the merging of peaks, peak width or apparent shifting of peaks. When atomic models are available, techniques such as Reverse Monte Carlo (RMC) can be used to create precise matches between simulation and experiment.^[24]^ With a strictly atomic model, RMC could preferentially adjust coordinates of atoms at the edges of crystallites to capture changes in the RDF due to configurational disorder. In HDRDF, we can imagine an analogous procedure in which RMC adjusts the coordinates of the atomic models for each phase, while leaving the mesoscale contribution intact.

## 3 Conclusion

The neutron and X‐ray scattering data of the lignin‐based carbon composites (LBCCs) generated by Tenhaeff et al. and García‐Negrón et al. respectively were successfully modeled using HDRDF 3 and granted both quantitative and qualitative understandings of the complex material structure in addition to the identification of nanoparticle shape. With the aid of HDRDF 3, trends in PSPP relationships were identified as increasing crystallite size, crystalline volume fraction, and composite density as well as the transformation from spherical crystalline particles to ellipsoids as reduction temperature was increased and the composites became more graphitic in nature. Through modelling with HDRDF 3 it was found that the amorphous carbon phase of SG reduced at 1050 °C is less dense compared to other feedstocks and for all feedstocks the nanoscale composite density of LBCCs increases with increasing reduction temperature. The average interplanar distance in crystallites was found to be 3.44 nm for all feedstocks at all reduction temperatures except for KSW samples which had an interplanar distance of 3.35 nm, like that of AB stacked graphite. Through a combination of modelling with HDRDF 3 and visual analysis of HR‐TEM images, the crystalline volume fraction was determined to increase with increasing reduction temperature for all feedstocks which become partially graphitic at a reduction temperature of 2000 °C. The crystalline volume fraction varied between 15–20 % for feedstocks reduced at 1050 °C and 40–45 % for feedstocks reduced at 1500 °C. The transition from spherical to ellipsoidal particle shapes as reduction temperature was increased from 1050 to 1500 °C was attributed to the adoption of amorphous graphene particles into the crystalline nanoparticles. It is also suggested that the higher oxygen content found in the KSW and SG samples is due to higher amounts of ether linkages that persisted through pyrolysis. The ether linkages that survived pyrolysis acted as a scaffold, providing structure for crystallites to grow into larger graphitic structures more rapidly. Further, inspection of the HR‐TEM of kraft softwood and switchgrass reduced at 2000 °C suggests that the large rod‐like crystallites could be multiwalled carbon nanotubes.

The HDRDF 3 software can now be used on parallel architectures and allows models with arbitrary domain geometries. Structural parameters are optimized via conjugate gradient optimization and crystalline/amorphous domain shapes can be identified via least‐error analysis, greatly reducing the human time, effort, and error of hand‐eye fitting that was present in previous models. HDRDF 3 achieved a reduction in computational cost of five orders of magnitude compared to molecular dynamics simulations of these LBCCs. HDRDF 3 can now be considered a generalized physics‐based tractable model for rapid modelling and understanding of the local structure of complex composite materials with only a small computational cost. Plans for future updates involve modules for including crystalline and amorphous polydispersity, customizable states of porosity in the mesoscale model as well as multiple crystalline and amorphous phases.

The quantitative results on LBCC structure from this work are being used to direct current work on optimizing LBCCs for use as sustainable hard carbon anodes in lithium and sodium ion batteries.

## Experimental Section

### Data Collection

The RDF data for this work was gathered at room temperature from the 11‐ID−B beamline at The Advanced Photon Source (APS) with 0.2113 Å wavelength. For the hardwood, pine, and switchgrass materials, lignin was extracted from the plant matter via the organosolv process.^[25]^ The kraft softwood lignin was created through the kraft process.^[26]^ The lignin feedstocks were carbonized according to the procedure of García‐Negrón et al., with reduction temperatures of 1050, 1500, and 2000 °C for each feedstock.[^4a^, ^9^] Samples were prepared in capillaries in triplicates for the scattering experiments to account for possible sample inhomogeneity. The RDF, or *g(r)*, for each sample were calculated from the X‐ray scattering data with the xPDFsuite software with lower and upper limits on the Fourier transform integral of 0.1 and 22.0 Å^−1^, respectively and a value of 0.8 for the polynomial smoothing function (*rpoly*).^[27]^ Fourier ripples are a result of the Fourier transformation from reciprocal space to real space and are considered noise in the experimental data. The Fourier ripples arise as artificial peaks in low *r* and long scale oscillations in high *r*. These ripples have been removed for *r<3.0* Å in our experimental data as to not introduce a significant source of error when the experimental and modeled RDFs are compared during the structural feature optimization step of HDRDF.

The neutron scattering data was gathered by McNutt et al. at the Nanoscale‐Ordered Materials Diffractometer (NOMAD) beamline at the Spallation Neutron Source (SNS) at Oak Ridge National Laboratory (ORNL).^[13a]^ The three LBCC samples were loaded in quartz capillaries and placed in the beamline for 2 h with an argon atmosphere at room temperature. Scattering from a solid vanadium rod provided data for background subtraction and normalization. RDFs were obtained by Fourier transform of S(Q) with Q_max_ of 30 Å.

### High Resolution Transmission Electron Microscopy (HR‐TEM)

HR‐TEM was used to gain visual evidence and qualitative information of the effects of feedstock and reduction temperature on the local structure of lignin‐based carbon composites. The lignin‐based carbon composites were first powdered and mixed with methanol at 0.2 : 99.8 wt%. To achieve separation of particles the mixture was then sonicated for 20 minutes. The mixture was then pipetted onto the surface of a lacey carbon 200 mesh copper grid (Cat. # LC200‐CU) as specified for TEM from EMS. Images were gathered using the Libra 200 MC with an accelerating voltage of 200 kV and vacuum pressure lower than 2.0×10^−6^ mbar.

### Hierarchical Decomposition of the RDF

The hierarchical decomposition of the RDF occurs in stages with the first stage separating phases of a complex material. For a composite composed of two phases, labeled a for amorphous and c for crystalline, total RDF, $g_{tot}$ , can be expressed at the first level of the decomposition as linear combination of the pair‐wise components, $g_{aa}$ , $g_{cc}$ , and $g_{ac}=g_{ca}$ , weighted by the relative atom fractions, $x_{a}$ and $x_{c}$ [Eq. 1]:

(1) $$g_{tot}\left(r\right)=x_{a}^{2}g_{aa}\left(r\right)+2x_{a}x_{c}g_{ac}\left(r\right)+x_{c}^{2}g_{cc}\left(r\right)$$

Subsequent stages of decomposition occur to a point at which each component of the RDF can be represented with a tractable physics‐based model. A detailed and rigorous explanation of the hierarchal decomposition theory is available in works by Oyedele et al. and García‐Negrón et al.[^17^, ^18^] In this implementation of HDRDF, the following procedure is adopted. For RDF components representing scattering by atoms within the same phase, the second level of decomposition is into atomistic and mesoscale components [Eq. (2 and 3]:

(2) $$g_{aa}\left(r\right)=g_{aa}^{atom}\left(r\right)+g_{aa}^{meso}\left(r\right)$$

(3) $$g_{cc}\left(r\right)=g_{cc}^{atom}\left(r\right)+g_{cc}^{meso}\left(r\right)$$

For RDF components representing scattering by atoms within different phases, the second level of decomposition is strictly a mesoscale component [Eq. 4]:

(4) $$g_{ac}\left(r\right)=g_{ac}^{meso}\left(r\right)$$

The practical motivation for this choice of decomposition has two origins. First, previously published molecular simulation work on LBCCs has associated all sharp peaks with features arising from pairs of atoms contained within a single graphitic crystallite in the crystalline domain or a single graphene fragment in the amorphous domain.^[13a]^ These contributions fall within $g_{aa}$ and $g_{cc}$ . Second, static models of the graphitic crystallites or graphene fragments are readily generated from existing crystal structure databases; therefore, the atomic contribution is tractable. The same degree of catalogued knowledge does not extend to the interfaces, making an atomic model for $g_{ac}$ a more suitable topic for the more computationally intensive molecular simulation approach. Fortunately, for the materials studied here, the empirical evidence supports this level of decomposition.

**Figure 6:**
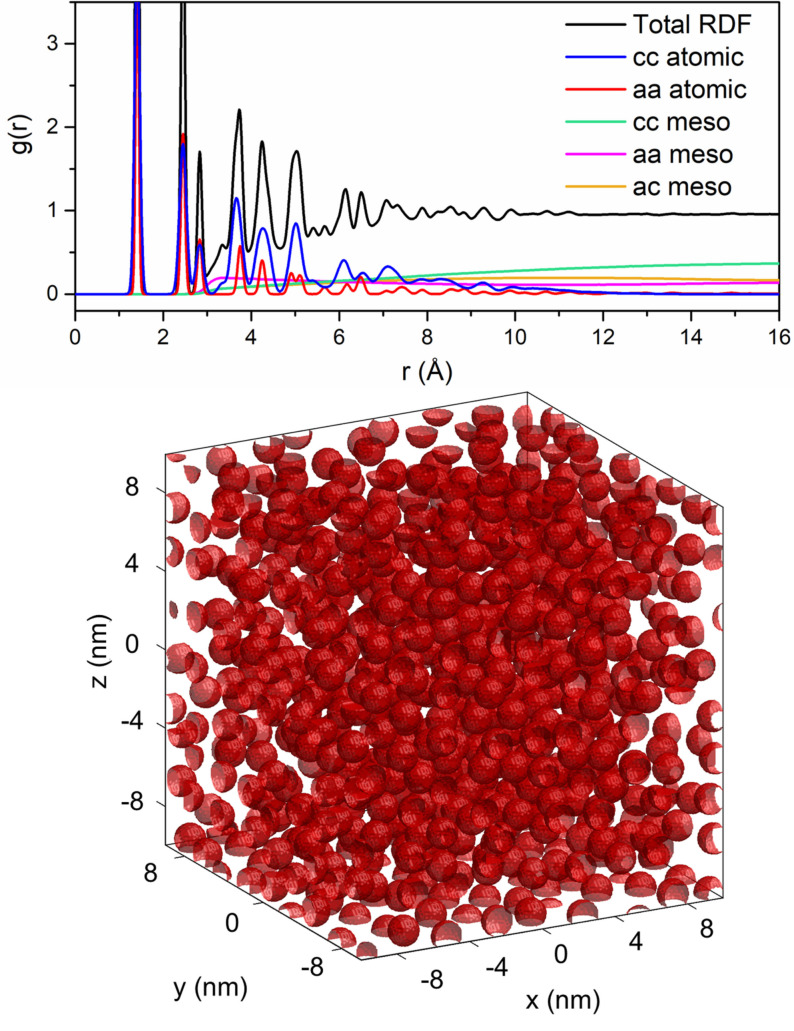
Hierarchical decomposition of the RDF with components 1) cc atomic: atomic crystalline intraparticle, 2) aa atomic: atomic amorphous intraparticle, 3) cc meso: mesoscale crystallite interparticle, 4) aa meso: mesoscale amorphous interparticle, 5) ac meso: mesoscale crystalline‐amorphous interparticle (Top).Mesoscale model with 50 % crystalline volume fraction and 1.5 nm diameter spherical crystallites (red) and an encapsulating amorphous matrix (white) (Bottom).

Specifically, the five components of the decomposition are 1) discrete atomic contribution from pairs of atoms inside a crystallite, $g_{cc}^{atom}\left(r\right)$ , 2) discrete atomic contributions from pairs of atoms in the amorphous phase, $g_{aa}^{atom}\left(r\right)$ , 3) mesoscale contribution between pairs of crystallites, $g_{cc}^{meso}\left(r\right)$ , 4) mesoscale contribution between amorphous domains, $g_{aa}^{meso}\left(r\right)$ , and 5) mesoscale contribution between crystalline and amorphous domains, $g_{ac}^{meso}\left(r\right)$ . The total RDF is then calculated from a weighted sum of each component, where the weight for each component of the hierarchical decomposition of the RDF is determined by the component volume fraction and density of each phase and ensure that the total RDF converges to unity as the separation between atoms approaches infinity. Each of these contributions are detailed in Figure 6. In Figure 6, clearly sharp features arise from contributions to the RDF with atomic resolution, while broader features are associated with mesoscale components.

### Advances from Previous Implementations of HDRDF

The primary improvement in the current version of HDRDF is the discretization of the model at the mesoscale. As shown in Figure 6, the area enclosed within the red surfaces is designated as the crystalline phase and the contiguous area outside the red surfaces is designated as the amorphous phase. In previous works, analytical solutions were derived and employed to rapidly evaluate the six‐dimensional integral generating the mesoscale RDF between spherical crystallites and the four‐dimensional integral generating the mesoscale RDF between parallel circular fragments of graphene. The analytical elegance was not readily amenable to arbitrary crystallite shapes or even polydispersity of spheres. In HDRDF 3, the analytical solutions have been replaced with a fully spatially discretized model of the composite in which the multi‐dimensional integrals are evaluated via Monte Carlo (MC) integration. While stochastic integration is certainly more computationally demanding compared to evaluation of analytical functions, it still requires several orders of magnitude less computational resources than the alternative, which is molecular dynamics simulation. Moreover, numerical integration opens the door to modelling composites with arbitrary particle shape, orientation (for non‐spherical particles), polydispersity and mesoscale structure (e. g. crystallites distributed on an ordered lattice versus randomly distributed crystallites).

The spatial discretization also eliminated the need of creating empirical ways to deal with experimental data that was not well modeled by spherical crystallites as was necessary in previous efforts. The analytical approach worked well for composites when the crystalline volume fraction was low and the separation between particles high. However, when the crystalline volume fraction was high, the particles began to be packed together, resulting in a flat interface between two otherwise spherical crystallites. This geometry required a sharp increase in mesoscale crystalline‐crystalline component, not possible with the analytical solution. In previous versions of HDRDF, this feature in highly crystalline composites was modeled with a parameterized erfc function. This ad hoc approach is no longer necessary with the MC integration of a spatially discretized model.

As a minor note, the previous use of HDRDF to examine carbon composites contained a third level of decomposition, separating the atomic crystalline‐crystalline component into contributions arising from C atoms within the same plane and C atoms in two different planes of graphite.^[18]^ In this work, the graphitic nanocrystallite is represented as a single atomic structure. The ability to vary the d‐spacing in graphite is retained by allowing the c vector of the unit cell to vary.

### Insights from Mesoscale Contributions

**Figure 7:**
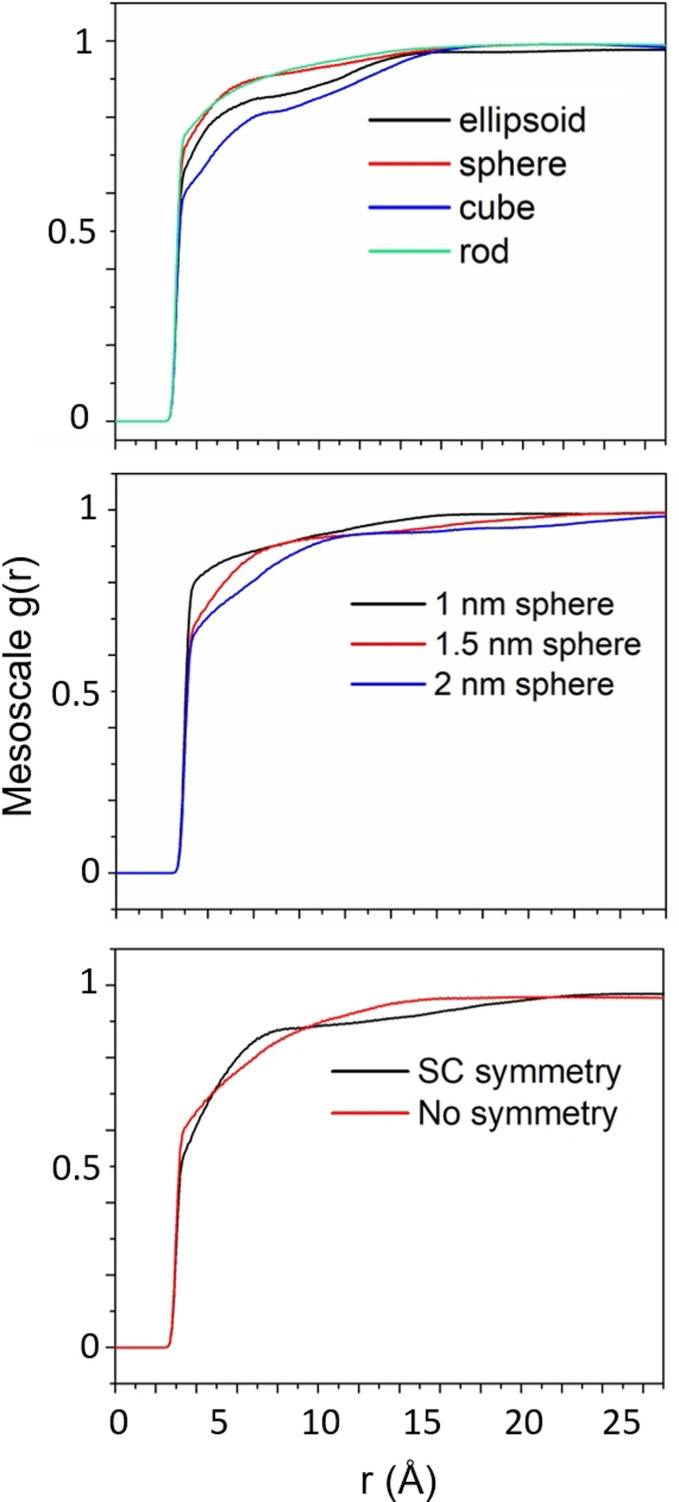
Intercrystallite mesoscale contributions, $g_{cc}^{meso}\left(r\right),\;$ to the total RDF aid in particle shape determination (top), particle size determination (middle), and mesoscale particle symmetry in the composite (bottom).

Radial distribution function features that define particle shape and size are difficult to determine when viewing a total RDF but are easily constructed with the HDRDF technique. The mesoscale contributions from the hierarchal decomposition play an important role in the identification of particle shape and size and in addition can aid in the determination of mesoscale symmetry of crystalline domains in composite materials. In Figure 7 below, various particle shapes, sizes and symmetry are shown with their corresponding intercrystallite mesoscale contributions, $g_{cc}^{meso}\left(r\right),\;$ to the total RDF. The plots in Figure 7 show the mesoscale intercrystallite contribution to the RDF for a set of similarly sized particle shapes, a set of differently sized crystallite nanospheres, and a set of simple cubic arranged nanospheres vs. randomly placed nanospheres (no symmetry). These plots are included to highlight the differences in the mesoscale contribution to the total RDF and show that the isolation and analysis of $g_{cc}^{meso}\left(r\right)$ can lead to qualitative and quantitative information when modelling sets of experimental samples. The mesoscale contributions are zero until after 3 Å since distances shorter than 3 Å are included in the discrete atomic contributions to the RDF.

### Model Creation and Optimization

In this section we describe the flow and methods of operation for the HDRDF 3 software. Crystalline phases (three dimensional volumes cut from a bulk graphite structure) and amorphous base units (represented by graphene fragments) are input into HDRDF with their respective particle shape, lattice vectors and angles, and fractional coordinates. To handle arbitrary geometries of crystalline and amorphous domains, HDRDF allows custom cartesian coordinate inputs. These atomic models are then used to compute the atomic contributions to the RDF from the crystalline and amorphous phases by constructing a histogram of all interatomic distances and applying gaussian type anisotropic thermal noise. Next, the crystallite particles are arranged in a 3‐dimensional structure according to user input (i. e., simple cubic formation, close packed, random placement, etc.) and the component‐wise volume fractions. The 3‐dimenstional mesoscale model is projected to a digitized 3‐d mesh with 0.2 Å resolution as shown in Figure 6. Sections of the mesh that are not defined with crystalline particles can be defined as an encapsulating amorphous matrix. The mesoscale model is a box whose size is generated to be greater than twice the length of the experimental RDF length used for comparison. This model sizing technique avoids artifacts in the modeled RDF that could arise by using a smaller mesoscale model with periodic boundary conditions. The mesoscale components of the RDF decomposition are then constructed with Monte Carlo integration performed on the digitized mesh where the number of sample points for each mesoscale contribution are based on component volume fraction and component density. The mesoscale components, $g^{meso}\left(r\right),\;$ are then linearly interpolated to the experimental resolution (usually 0.01 Å) and the total RDF is formed from the weighted sum of the atomic and mesoscale contributions as seen in Figure 6. The total modeled RDF is then compared to experiment and a least‐squares error is calculated to measure goodness of fit. Iterative optimization of structural parameters is then carried out via BFGS conjugate gradient method until the specified convergence criteria are met.^[28]^

### HDRDF Output

After convergence of the iterative optimization, HDRDF outputs the optimized structural parameters as well as the total modeled RDF and each component of the hierarchical decomposition. In addition, there are options to allow HDRDF to output the crystalline, amorphous, and mesoscale 3D models for visualization.

### Previous Versions of HDRDF

The first iteration of this method developed by Oyedele et al. used analytical solutions to six‐dimensional integration and could only be employed for spherical crystallites due to the difficulty of complex integration over arbitrary geometries. The first application of this method was used to successfully model both the total neutron scattering (NS) RDF of a carbon‐composite and on a component‐by‐component basis against MD models of the aforementioned carbon‐composites.[^13a^, ^17^] The second generation of the hierarchical decomposition method was developed in MATLAB by García‐Negrón et al. and was implemented on a series of three hardwood‐lignin‐based carbon‐composites (LBCCs) with increasing reduction temperature.^[18]^ García‐Negrón's model allowed iterative by‐hand optimization of structural parameters such as crystallite domain size, crystalline and amorphous volume fractions, and density.^[18]^ Modeled RDFs were compared on a component‐by‐component basis versus three lignin‐based carbon composite MD models of 10, 50, and 90 % crystallinity which emulated the carbon‐composites for hardwood lignin pyrolyzed and reduced at 1050, 1500, and 2000 °C respectively.[^13a^, ^18^] This second implementation of the hierarchical decomposition method maintained the reduction in computational cost by six orders of magnitude compared to the computational cost in obtaining the modeled RDF via MD simulation.

## Note

Mention of trade names or commercial products in this article is solely for the purpose of providing specific information and does not imply recommendation or endorsement by the U. S. Department of Agriculture. USDA is an equal opportunity provider and employer.

## Conflict of interest

The authors declare no conflict of interest.

4

## Supporting information

As a service to our authors and readers, this journal provides supporting information supplied by the authors. Such materials are peer reviewed and may be re‐organized for online delivery, but are not copy‐edited or typeset. Technical support issues arising from supporting information (other than missing files) should be addressed to the authors.

Supporting Information

## Data Availability

The data that support the findings of this study are available from the corresponding author upon reasonable request.
