# Supplementary material for: Local Structure Analysis and Modelling of Lignin‐Based Carbon Composites through the Hierarchical Decomposition of the Radial Distribution Function
Source: ChemistryOpen. 2022 Feb 17;11(2):e202100220. doi: 10.1002/open.202100220 (PMC8850997; doi:10.1002/open.202100220)
Supplement: Supplementary file 1 — Supporting Information [file OPEN-11-e202100220-s001.pdf]

Author Contributions

V.G.-N. Data curation:Supporting; Writing – review & editing:Supporting; Data Aquisition:Equal
D.H. Conceptualization:Supporting; Funding acquisition:Lead; Investigation:Supporting; Project administration:Lead; Supervision:Lead; Writing – review & editing:Supporting
D.K. Conceptualization:Equal; Data curation:Supporting; Formal analysis:Supporting; Funding acquisition:Lead; Investigation:Supporting; Methodology:Supporting; Project administration:Lead; Supervision:Lead; Validation:Supporting; Writing – review & editing:Equal
